# Plastic and rubber polymers in urban PM_10_ by pyrolysis–gas chromatography–mass spectrometry

**DOI:** 10.1007/s00216-025-05906-z

**Published:** 2025-05-13

**Authors:** Tatu Martinmäki, Sanna Saarikoski, Hilkka Timonen, Jarkko V. Niemi, Markus Sillanpää

**Affiliations:** 1 Finnish Environment Institute, Research Infrastructure, Mustialankatu 3, 00790 Helsinki, Finland; 2 Department of Chemistry, University of Helsinki, P.O. BOX 55, 00014 Helsinki, Finland; 3 Finnish Meteorological Institute, 00560 Helsinki, Finland; 4 Helsinki Region Environmental Services Authority, 00240 Helsinki, Finland

**Keywords:** Tyre and road wear particles, Aerosol, Air pollution, Nanoplastics

## Abstract

Microplastics, including tyre and road wear particles, have been detected in every environmental compartment in both urban and remote areas. However, their contribution to atmospheric particulate matter is still sparsely explored. These airborne micro- and nanosized particles are continuously inhaled and pose risks to the environment and public health. The objectives of this study were to develop and validate a thermoanalytical method for the quantification of microplastics in urban particulate matter. Aerosol particles smaller than 10 µm in aerodynamic diameter (PM_10_) were sampled at the kerbside in Helsinki, Finland, during spring 2024. The samples were pretreated by homogenization and thermal desorption prior to chemical analysis by micro-furnace pyrolysis–gas chromatography–mass spectrometry. The developed method was validated in terms of selectivity, limits of quantification, linear range, trueness, precision, and measurement uncertainty. Instrument quantification levels were 8–270 ng. Expanded measurement uncertainties were 25–30% and 50–70% for the studied tyre wear rubbers and thermoplastics, respectively. Polyethylene, polyethylene terephthalate, polypropylene, polystyrene, and tyre and road wear particles were detected in urban PM_10_ samples, and their sum accounts for 1–3% of total PM_10_. These results represent the level of airborne microplastic particles to which people can be exposed in urban environments.

**Graphical Abstract:**

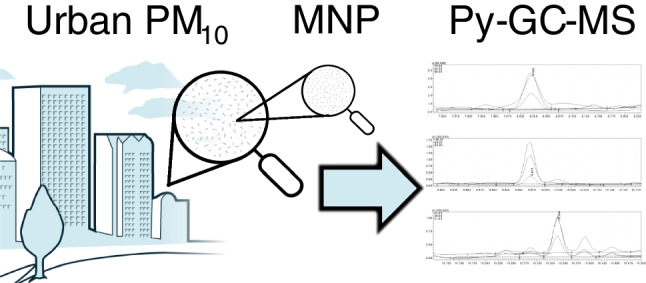

**Supplementary Information:**

The online version contains supplementary material available at 10.1007/s00216-025-05906-z.

## Introduction

Global plastic production has increased annually since the 1950 s [1] and reached 4.1 × 10^11^ kg in 2023 [2]. Plastic pollution is considered one of the emerging environmental challenges due to its ubiquitous and persistent nature [3]. Micro- and nanoplastics (MNP) are defined as synthetic polymeric particles with sizes ranging from 1 to 1000 µm and 1 to 1000 nm, respectively [4]. Tyre and road wear particles (TRWP), generated by vehicle tyre abrasion, have been recently included in the definition of MNP, and are considered one of the main contributors to environmental MNP [5]. MNP are known to contain a variety of harmful chemicals, such as UV-blockers, plasticizers, and fire retardants, and can also act as carriers for substances in the environment due to their high surface area and suitable chemical qualities [6]. Additionally, there are associated health hazards for humans [7] and risks in both aqueous [8, 9] and terrestrial [10–12] environments.

Environmental microplastic studies are typically based on vibrational spectroscopy, i.e. micro-infrared or Raman spectroscopy [13]. These techniques are not suited for the identification of particles smaller than 1 µm in diameter, or strongly absorptive TRWP [14]. Recently, pyrolysis gas–chromatography–mass spectrometry (Py-GC–MS) has been applied for qualitative and quantitative analysis of MNP in various environmental matrices, such as waters and soils [13]. A principal difference between the techniques used in environmental MNP studies is that methods based on vibrational spectroscopy provide number-based results, whereas Py-GC–MS provides mass-based results.

Micro- and nanoplastics have been detected and studied in every environmental compartment, drinking water, and foodstuff, but the exposure of humans to airborne MNP in ambient air is unknown. Airborne MNP have been previously reported in urban areas [15–18] and in the northern Atlantic air [19]. PM_10_ is defined as aerosol particulate matter smaller than 10 µm in diameter [20, 21]. Particles in this size range may penetrate beyond the larynx and have been reported to cause adverse effects on human health, including respiratory symptoms and cardiovascular diseases [22]. Furthermore, there is an association between urban air quality and increased mortality [23]. While TRWP are estimated to contribute a few percent to ambient air pollution [24, 25], the contribution of MNP to PM_10_ has yet to be determined.

Considering environmental MNP studies in general, the quality of the measurements should be rapidly addressed to increase transparency, reproducibility, and comparability of the results, as discussed recently by Cowger et al. [26]. It is crucial to understand the abundance and composition of particulates released into the environment throughout society to make effective political decisions and to monitor the effects of the upcoming emission standards, i.e. Euro 7 in 2026 [27].

The aim of the study was to develop and validate a sensitive and straightforward micro-furnace Py-GC–MS method for the quantification of thermoplastics polyethylene (PE), polyethylene terephthalate (PET), polypropylene (PP), and polystyrene (PS), which are among the most produced worldwide [2], and prevalent tyre wear rubbers natural rubber (NR) and styrene-butadiene rubber (SBR) in kerbside PM_10_. The presented airborne MNP mass concentrations represent the levels to which people are exposed in urban canyons. To our knowledge, this is the first study to present the key validation parameters of a quantitative Py-GC–MS method for airborne MNP and the contribution of airborne MNP to urban PM_10_.

## Experimental

### Chemicals and reagents

Analysis grade trichloromethane was purchased from Supelco (St. Louis, USA). Poly(styrene-d8) (product number P18459-dPS), deuterated poly(1,4-isoprene-d7) (P43125-d7Ip), and poly([deuterated styrene-d8]–co–[deuterated butadiene-d6]) random co-polymer (P19280-dPSdPBdran) were purchased from Polymer Source, Inc. (Dorval, Canada). Tetramethylammonium hydroxide 25% in methanol (TMAH) and cis-polyisoprene were purchased from Sigma-Aldrich (St. Louis, USA). Microplastics calibration standard set (MPs-SiO_2_) and Diluent SiO_2_ powder were purchased from Frontier Laboratories Ltd. (Fukushima, Japan). PE and PP granulates from Borealis Polymers (Kulloo, Finland), PS and SBR granulate from Sigma-Aldrich, and PET bottle were cryo-milled and used in method development.

### Kerbside PM_10_ sampling

**Fig. 1:**
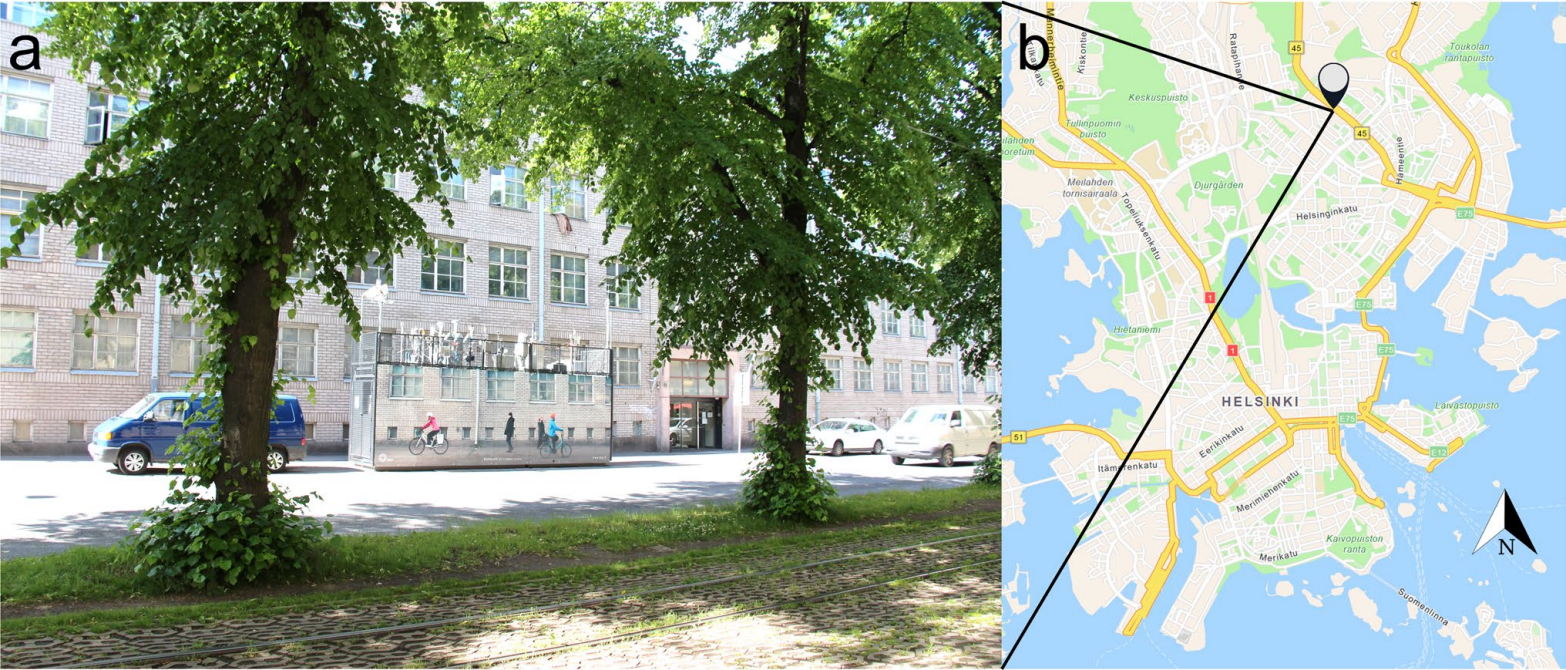
**a** Photo of the sampling location; the sampling container is located at the centre of the photograph. **b** The sampling location is indicated by the black pinpoint, and orientation to north (N) is indicated by the arrow at the bottom right corner of the map

Aerosol samples were collected at Mäkelänkatu air quality supersite, Helsinki, Finland (60.196532, 24.951670), during April–May 2024 (Fig. 1) [28]. This kerbside sampling station is next to a six-lane street with a speed limit of 40 km h^−1^, and it is located 4 km north of Helsinki city centre. According to the statistics provided by the City of Helsinki, 18,000 vehicles on average were daily passing the urban canyon 42 m in width and surrounded by 17-m-tall buildings. The sampling took place at a height of 4 m from ground level. Aerosol sampling equipment consisted of an aluminium PM_10_-inlet (Digitel Elektronik AG, Volketswil, Switzerland) connected to a stainless-steel filter cartridge (Gelman Sciences Inc., Ann Arbor, MI, USA) with stainless-steel tubing. The samples were collected on calcined quartz fibre filters (Pallflex Tissuquartz 2500-QAT-UP, 47 mm, Pall Life Sciences, USA) at a sampling flow rate of 38.3 L min^−1^. Flow was monitored with a mass flow meter. The sampling volumes varied between 320 and 770 m^3^. Total PM_10_ was measured with a continuous particulate matter monitor (Fidas 200, Palas GmbH, Karlsruhe, Germany). Details of the sampling times and volumes are presented in Table S2. Field blanks were prepared identically to PM_10_ samples apart from the sampling volume (0.11 m^3^).

### Sample pretreatment

Quartz fibre filters were weighed and homogenized with a ball mill at room temperature using two 30-s cycles at 30 Hz, with a 5-min cool-down period between the cycles. Stainless steel vessels and stainless-steel milling balls (Retsch) were used. Subsamples of 8–14 mg were weighed (Mettler Toledo XP 56, Columbus, OH, USA) accurately into pyrolysis cups (Eco-Cup LF PY1-EC80 F, Frontier Laboratories Ltd., Fukushima, Japan). Field blanks and empty pyrolysis cups as laboratory blanks were treated as samples to estimate possible contamination during sample pretreatment. Three deuterated internal standards, poly(styrene-d8), poly(1,4-isoprene-d7), and poly([styrene-d8]–co–[butadiene-d6]) random co-polymer, were dissolved in trichloromethane at concentrations of 10 µg mL^−1^, 60 µg mL^−1^, and 36 µg mL^−1^, respectively. Ten microlitres of internal standard solution was added to the pyrolysis cups with the sample. Pyrolysis cups were heated in a chamber furnace (Nabertherm N11/H, Lilienthal, Germany) to 200 °C for 10 min. For enhanced PET detection [29], calcined quartz fibre filter disc (Ø 4 mm) was impregnated with 10 µL of tetramethylammonium hydroxide and placed on top of the sample cups. In addition to enabling reactive pyrolysis with TMAH, the purpose of the filter disc was to ensure that the pulverized sample remains in the pyrolysis cup during the analysis and to increase the SiO_2_ matrix in the pyrolysis cup.

### Py-GC–MS analysis and application to kerbside PM_10_ samples

Instrumentation consisted of a Multi-Shot Micro-Furnace Pyrolyzer (EGA/PY-3030D, Frontier Laboratories Ltd.) with an Auto-Shot Sampler (AS-2020E, Frontier Laboratories Ltd.) coupled to a GC–MS (GCMS-QP2020 NX, Shimadzu, Kyoto, Japan). The GC was equipped with a precolumn (UA 50-1 M, Frontier Laboratories Ltd.), an analytical column (UA5-30 M 0.5 F, Frontier Laboratories Ltd.), and vent-free adapter (Frontier Laboratories Ltd.). The temperature and time of single-shot pyrolysis were set to 600 °C and 0.1 min. The temperatures of the pyrolyser interface and the GC inlet were 300 °C. The system was run in a constant flow mode of 1.0 mL min^−1^ with a split ratio of 1:25 and a 3.0-min solvent delay. Ultrapure helium (≥ 99.9995%) was used as the carrier gas. MS ion source and interface temperatures were 200 °C and 300 °C, respectively. The thermal gradient in the GC was as follows: an initial 3-min hold at 40 °C was followed by a temperature increase at a rate of 20 °C min^−1^, a 6-min hold at 280 °C, a temperature increase at a rate of 25 °C min^−1^, and a final hold at 310 °C for 20 min. Single ion monitoring (SIM) mode was used for data acquisition with an event time of 0.2 s for each SIM window. An electron ionization (EI) source was used with 70-eV ionization energy. Data acquisition and analysis were performed in GCMSsolution software (version 4.54, Shimadzu).

MPs-SiO_2_ was diluted with Diluent SiO_2_ powder and homogenized with a ball mill (Retsch 400 MM, Haan, Germany) for the calibration of the instrument (standard dilution). Another dilution was prepared for spiking the control samples in validation studies (control dilution). Five standard solutions (2.5, 5.0, 10, 20, and 50 µg mL^−1^) and one control solution (10 µg mL^−1^) of cis-polyisoprene in trichloromethane were prepared for the calibration of the instrument and spiking the control samples in the validation study, respectively. Details of the prepared dilutions and solutions used throughout the study are presented in Table S1.

For quantification of the polymers in PM_10_ samples, the instrument was calibrated by adding the standard dilution and cis-polyisoprene in trichloromethane to the pyrolysis cups. A linear fit and five to six calibration levels were used for each polymer. Weighing of the linear fit was applied to increase the accuracy of the linear fit at low concentration points. Aerosol samples were analysed in triplicates. Calibration standards, blanks, and samples were pretreated according to the section “Sample pretreatment”. Details of the calibration standard levels, blanks, and samples are presented in Table S2.

Polymer concentrations in air ($c\left(polymer\right)$) are calculated in Eq. 1.

1 $$\begin{array}{c}c\left(polymer\right)=\frac{m\left(polymer\right)\;\times\;m\left(tot\right)}{m\left(sub\right)\;\times\;V\left(sample\right)}\\\end{array}$$

where $m\left(tot\right)$ is the total mass of filter after the sampling, $m\left(sub\right)$ the subsample mass of homogenized sample, $m\left(polymer\right)$ is the determined mass of the polymer in the sub sample, and $V\left(sample\right)$ is the sampling volume. Field blank concentrations were subtracted from the samples.

Tyre wear rubber concentrations in air were converted to TRWP concentrations ${c}_{TRWP}$ by Eq. 2, according to ISO/TS 20593:2017 [30].

2 $$\begin{array}{c}{c}_{TRWP}=\frac{{c}_{SBR}\;\times\;\frac{1-{S}_{c}}{1-{S}_{t}}+\;{c}_{NR}}{{F}_{r}\;\times\;{F}_{T}}\end{array}$$

where ${c}_{SBR}$ is the determined SBR concentration in air, ${S}_{c}$ is the styrene content of SBR used in the calibration curve (0.235), ${S}_{t}$ is the market share average styrene content in tyre tread (0.15), ${c}_{NR}$ is the determined NR concentration in air, ${F}_{r}$ is the rubber fraction in passenger and truck tread (0.5), and ${F}_{T}$ is the total fraction of passenger and truck tread in TRWP (0.5).

### Method validation

Method selectivity, limits of quantification, and linear range were determined in a validation study by adding ten levels of standard dilution and cis-polyisoprene in trichloromethane to the pyrolysis cup. The levels were spread evenly throughout the estimated linear area. An empty pyrolysis cup and two blank samples with added internal standard were prepared and analysed. Method selectivity was determined by observing the signals generated from the selected pyrolysates and ions in the blank samples within the validation studies. The low limit of quantification (LLOQ) and high limit of quantification (HLOQ) levels for each pyrolysate were determined by observing the signal levels of the selected pyrolysates and ions for quantification in the validation study. For the LLOQ, the signal of the ion used for quantification had a signal-to-noise ratio of larger than 10. For the HLOQ, the highest tested level prior to the detector saturation was selected. The proportion of variation in the linear fit was determined by the coefficient of determination *R*^2^. The linearity of the method was determined by residual analysis after the weighing of the linear fit. Blank samples included throughout the validation series were negative for the selected pyrolysates and ions of quantification and identification.

The trueness and precision of the developed method were determined by a recovery study. The instrument was calibrated by adding seven levels of standard dilution and five levels of cis-polyisoprene in trichloromethane to the pyrolysis cup. The calibration levels were spread evenly over the selected working range. Mid-calibration control samples were prepared by adding control dilution and cis-polyisoprene in trichloromethane to the pyrolysis cup. The control samples were analysed in five replicates. The study was repeated over three separate days, resulting in three calibration curves and 15 control samples analysed. All samples in the method validation study were pretreated as described in the section of “Sample pretreatment”, excluding the sample homogenization step.

Trueness $b\left(\%\right)$ of the method was calculated from results of the recovery study (*n* = 15) with Eq. 3.

3 $$\begin{array}{c}b\left(\%\right)= \frac{\overline{x }-{x}_{ref}}{{x}_{ref}}\times 100\end{array}$$

where $\overline{x }$ is the mean value of the spiked control samples and ${x}_{ref}$ is the calculated reference value.

The relative intermediate precision ${s}_{I}\left(\%\right)$ of the method was estimated by analysis of variance (one-way ANOVA) from the results of the recovery study by Eq. 4:

4 $${s}_{I}\left(\%\right)=\frac{\sqrt{\left(n-1\right)M{S}_{w}+M{S}_{b}}}{\overline{x}\sqrt{n} }\times 100$$

where ${MS}_{w}$ is the mean squares *within groups* in the ANOVA table, ${MS}_{b}$ the mean squares *between groups*, and $n$ the number of groups.

The combined standard uncertainty (${u}_{c}$) and the expanded measurement uncertainty ($U$) at 95% confidence level were calculated for each of the studied polymers with Eqs. 5 and 6, respectively.

5 $$\begin{array}{c}{u}_{c}=\sqrt{{{s}_{I}\left(\%\right)}^{2}+{b\left(\%\right)}^{2}}\end{array}$$

6 $$\begin{array}{c}U=2\times {u}_{c}\end{array}$$

### Quality assurance and quality control

To avoid contamination, samples, field blanks, laboratory blanks, and calibration standards were pretreated in a laminar flow cabinet or fume hood in a microplastic laboratory with HEPA-filtrated ventilation, when applicable. Cotton clothing and cotton lab coats were used during sample pretreatment. Prior to their use, glassware was cleaned with ultrapure water (18.2 MΩ·cm at 25 °C, filtrated with 0.22-µm polyethersulfone filter) and trichloromethane. All reagents and dilutions were analysed to rule out MNP contamination. Calcined and solvent-washed pyrolysis cups were used throughout the study. Steel equipment, i.e. tweezers, spatulas, and sample trays, were calcined prior to their use. Samples were stored in Pyrex glass petri dishes or folded aluminium foil when transferred from the sampling station or within the laboratory.

Blanks of three different levels were used throughout the study to estimate contamination of the samples from different stages of the process. Field blanks were collected on quartz fibre filters with a sampling volume of 0.11 m^3^. They were processed as samples and indicate the MNP contamination occurring during sampling, transport, storage, and sample pretreatment. Laboratory blanks went through all sample pretreatment steps except for the homogenization. Instrument blanks were analysed without pyrolysis cups to estimate the contamination of the analytical instrument.

## Results and discussion

### Polymer identification and quantification

The identification of the studied polymers by corresponding pyrolysates was based on the analyses of the standard materials. Considering the ions selected for quantification, a larger mass-to-charge ratio (m/z) generally provided better selectivity at the cost of sensitivity. The matrix and its possible interferences must also be considered when selecting the pyrolysates and corresponding ions. For example, PS was quantified in the present study by its trimer pyrolysate (2,4,6-triphenyl-1-hexene) due to its specificity and the method conditions, even though styrene would result in higher sensitivity with limited specificity. A summary of the pyrolysates and ions selected for identification and quantification of the studied plastic and rubber polymers is presented in Table 1.

**Table 1:** Pyrolysates, corresponding ions, and retention times of the studied plastic or rubber polymers used throughout the present study. The pyrolysate and ion used for quantification of the polymer are bolded

Plastic/rubber (abbreviation)	Pyrolysate	m/z	Retention time (min)	Internal standard
Polyethylene (PE)	**1-Eicosene**	**97**, 57, 83, 111	15.9	Poly(styrene-d8)
	1,20-Heneicosadiene	82, 55, 96	16.7	
	1-Octadecene	83, 97, 111	14.8	
	1-Nonadecene	83, 97, 111	15.3	
Polyethylene terephthalate (PET)	**Dimethyl terephthalate**	**163**, 194, 103	13.6	Poly(styrene-d8)
Polypropylene (PP)	**2,4,6-Trimethyl-1-nonene**	**43**, 29, 41	10.1	Poly(styrene-d8)
	2,4-Dimethyl-1-heptene	71, 57, 85	7.9	
Polystyrene (PS)	**2,4,6-Triphenyl-1-hexene**	**91**, 117, 312	20.9	Poly(styrene-d8)
	Styrene	104, 78, 51, 158	8.7	
Natural rubber (NR)	**Dipentene**	**136**, 93, 68	9.9	Poly(1,4-isoprene-d7)
	Isoprene	67, 53, 39	3.4	
Styrene-butadiene rubber/butadiene rubber (SBR/BR)	**4-Vinylcyclohexene**	54, **79**, 66	7.8	Poly([deuterated styrene-d8]–co–[deuterated butadiene-d6]), random
	4-Phenylcyclohexene	104, 158, 78, 91	12.4	
Poly([deuterated styrene-d8]–co–[deuterated butadiene-d6]), random (SBR-d14)	**4-Vinylcyclohexene-d12**	**86**, 60, 88	7.60	–
Poly(1,4-isoprene-d7) (PI-d7)	**Dipentene-d14**	**100**, 76, 117	9.65	–
Poly(styrene-d8) (PS-d8)	**2,4,6-Triphenyl-1-hexene-d24**	**98**, 126	20.60	–

The optimal pyrolysates used in the present study were mainly similar to those published in previous studies on airborne MNP. In contrast, Mizuguchi et al. [16] and Goßmann et al. [19] used 2,4-dimethyl-1-heptene and 4-phenylcyclohexene for PP and SBR, respectively, while Torres-Agullo et al. [31] selected 1,20-heneicosadiene and 2,4-dimethyl-1-heptene for PE and PP, respectively. In the present study, 1-eicosene for quantification of PE was chosen due to higher sensitivity with adequate selectivity over alternative pyrolysates, while 2,4,6-trimethyl-1-nonene and 4-vinylcyclohexene resulted in low detection limits and negligible interfering signals for the quantification of PP and SBR, respectively.

Polymers reported in previous studies, i.e. polyvinyl chloride, Nylon 6 and 66, acrylonitrile butadiene styrene, and polymethyl methacrylate, were excluded from this study due to the lack of specificity of the pyrolysates (naphthalene) or the sensitivity of the method at the studied levels (cyclopentanone and epsilon-caprolactam, 2-phenethyl-4-phenylpent-4-enenitrile, and methylmethacrylate).

### Method performance characteristics

The method proved to be linear throughout the tested range for PE, PP, PS, SBR, and NR. In contrast, PET signals did not follow the linear pattern. This can be explained by the fluctuating efficiency of derivatization at the studied levels, or by matrix effects originating from inorganic components in the sample [32]. Therefore, only qualitative results of PET in kerbside PM_10_ are discussed in the present study. The working range used to calibrate the instrument was selected according to the validation study by estimating the polymer concentrations in particulate matter samples. The LLOQs for the studied polymers were 8 to 270 ng, while the HLOQs were 500 to 8700 ng. The limit of detection (LOD) for PET varied between 140 and 380 ng in the validation study. The expanded measurement uncertainties at the 95% confidence level (U) were 25–30% for tyre wear rubbers (NR, SBR) and 50–70% for plastics (PE, PP, PS). The lowest combined measurement uncertainties were determined for polymers with corresponding deuterated internal standards, which emphasizes the importance of internal calibration in quantitative studies. Measurement uncertainties of analytical methods from previous studies on airborne MNP have not been reported. The performance characteristics of the developed method are summarized in Table 2. Detailed validation parameters are presented in Table S3.

**Table 2:** The performance characteristics of the developed method for the studied plastic or rubber polymers. Abbreviations: *LLOQ* lower limit of quantification, *HLOQ* higher limit of quantification, *u*_*c*_ determined combined measurement uncertainty, and *U* expanded measurement uncertainties

Polymer	Pyrolysate	LLOQ (ng)	HLOQ (ng)	Selected working range (ng)	${u}_{c}$(%)	U (%)
PE	1-Eicosene	270	8700	350–8700	25	50
PP	2,4,6-Trimethyl-1-nonene	70	2200	90–2200	33	70
PS	2,4,6-Triphenyl-1-hexene	8	4300	20–510	24	50
NR	Dipentene	25	6000	25–500	14	30
SBR	4-Vinylcyclohexene	28	7600	36–900	12	25

Mizuguchi et al. [16] determined LODs and coefficients of determination were comparable to our study: LODs ranged from single to tens of nanograms for the studied polymers and with *R*^2^ of linear fit > 0.9 for each of the polymers, respectively. Luo et al. [15] reported picogram-level limit of quantification (LOQ) for aerosolized polyethylene in their study. Goßmann et al. [19, 33] reported ≥ 0.9 coefficients of determination *R*^2^ for PP, SBR, and NR, also comparable with the present study.

### Micro- and nanoplastics in kerbside PM_10_

**Fig. 2:**
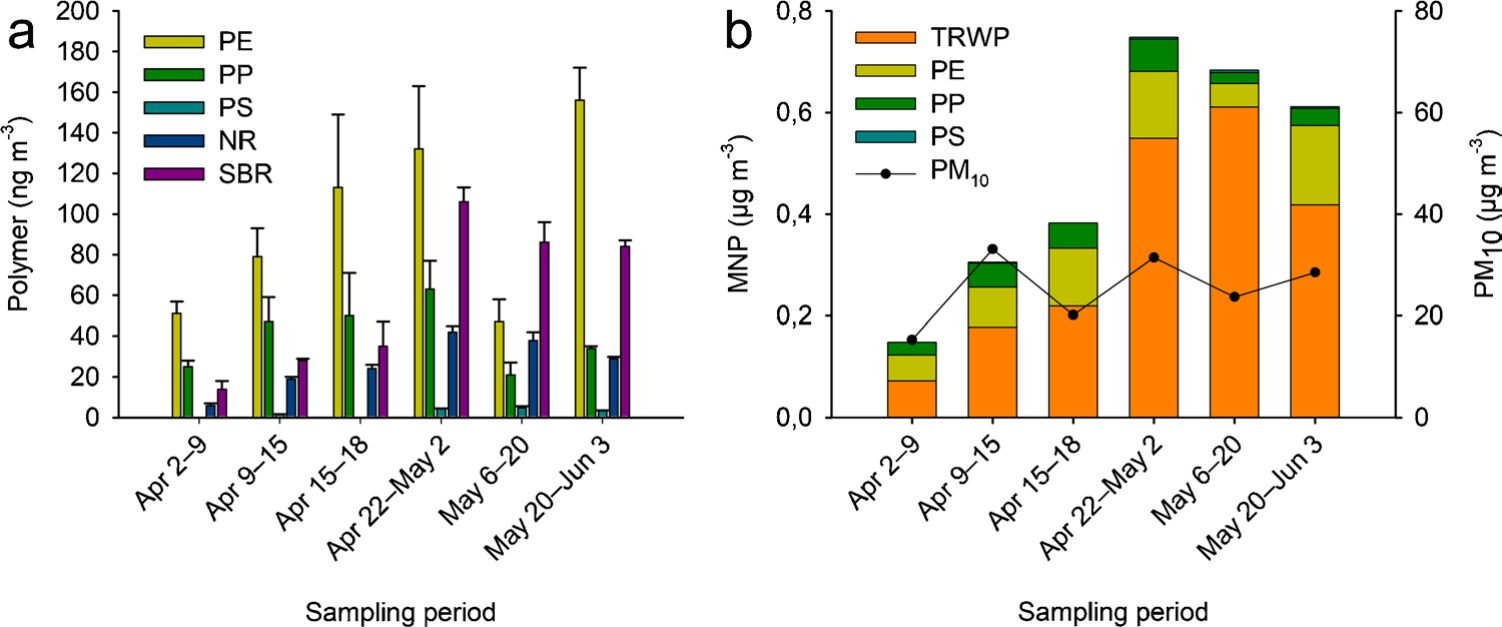
**a** Concentrations and standard deviations (*n* = 3) of plastic and rubber polymers in kerbside PM_10_. **b** MNP and total PM_10_ concentrations in kerbside PM_10_ samples collected in Helsinki, Finland, during spring 2024

The studied thermoplastics (PE, PP, PS, PET) and rubbers (NR, SBR) were detected in the collected filter samples during the sampling campaign. The studied polymers, excluding PS, were present in all the collected samples. While the polymer abundances fluctuated during the sampling campaign (Fig. 2a), the most abundant polymer in kerbside PM_10_ was PE, followed by PP, SBR, and NR, with concentrations of 47–156, 21–63, 14–106, and 6–42 ng m^−3^, respectively (Table 3). PS was detected in four out of six samples, and its concentrations were an order of magnitude lower than those of the other studied polymers. The sum of the measured plastic and rubber polymers accounts for 0.5–1.1% of PM_10_. While quantification of PET was not possible at the studied concentrations, the qualitative results confirm that PET fibrils or fragments are present also in the PM_10_ size fraction. Homogenization of the sample filter by ball-milling was successful, indicated by standard deviations of the sample replicates (Fig. 2a). Out of the two field blanks collected, field blank 2 showed signals for PE and PP slightly higher than the LLOQ. Details of the instrument calibration and blanks are presented in section S4.

**Table 3:** Concentrations ± standard deviations (*n* = 3) of plastic and rubber polymers and their relative contributions to PM_10_ in kerbside PM_10_ aerosol samples collected in Helsinki, Finland, during spring 2024

Sampling period	PM_10_ (µg m^−3^)	Concentration (ng m^−3^)									
		PE	PP	PS	NR	SBR	TRWP^1^	Sum of polymers	MNP^2^	TRWP-% PM_10_	MNP-% PM_10_
Apr 2–9	15	51 ± 6	25 ± 3	< LLOQ	6 ± 1	14 ± 4	72 ± 13	96 ± 8	168 ± 15	0.5	1.1
Apr 9–15	33	79 ± 14	47 ± 12	1.5 ± 0.2	19 ± 1	28 ± 1	177 ± 9	175 ± 18	352 ± 21	0.5	1.1
Apr 15–18	20	113 ± 36	50 ± 21	< LLOQ	24 ± 2	35 ± 12	219 ± 44	222 ± 43	441 ± 62	1.1	2.2
Apr 22–May 2	31	132 ± 31	63 ± 14	4.4 ± 0.2	42 ± 3	106 ± 7	549 ± 32	347 ± 35	896 ± 47	1.7	2.9
May 6–20	24	47 ± 11	21 ± 6	4.8 ± 0.8	38 ± 4	86 ± 10	461 ± 42	197 ± 17	658 ± 45	1.9	2.8
May 20–Jun 3	28	156 ± 16	34 ± 1	3.3 ± 0.2	29 ± 1	84 ± 3	418 ± 16	306 ± 16	725 ± 23	1.5	2.5

^1^Derived from NR and SBR concentrations following ISO/TS 20593:2017. Ambient air—determination of the mass concentration of tyre and road wear particles (TRWP)—pyrolysis-GC–MS method

^2^Sum of PE, PP, PS, and TRWP

The tyre wear polymer–derived TRWP concentrations, according to ISO/TS 20593:2017 [30], were 72–549 ng m^−3^. However, local variation of the tyre tread polymer composition and the use of winter tyres increase the uncertainty of this estimation. The studied MNP, consisting of PE, PP, PS, and tyre wear polymer–derived TRWP, accounted for 1–3% of PM_10_ (Fig. 2b). The increase of TRWP concentration at the middle of the sampling campaign can be explained by the snowmelt, which caused resuspension of road dust. Furthermore, the contribution of TRWP to PM_10_ in the present study (0.5–1.9%) corresponded to 0.7–1.2% for environments with population density > 3000 km^−2^ published previously by Panko et al. [24, 34].

There are two comparable studies published previously on airborne MNP smaller than 10 µm in the urban environment. The determined mass concentrations of plastic and rubber polymers in the present study are higher for PP, PS, and SBR than reported by Mizuguchi et al. (4.3 ng m^−3^, 1.2 ng m^−3^, and 1.4 ng m^−3^ for PP, PS, and SBR, respectively) in the PM_10_ size fraction [16]. Morioka et al. presented higher concentrations in the size fraction 0.43–11 µm (810 ng m^−3^ and 140 ng m^−3^ for PE and PS, respectively) but did not detect SBR [17]. Concerning the aerosol sampling, the size fraction and sampling volumes in the present study were comparable with Mizuguchi et al., while Morioka et al. multifractionated their sample with a lower sampling volume. A notable difference in the methodologies is that the present study used internal calibration, accounting for more precise quantification when compared to external calibration used in previous studies of MNP in urban ambient air. Also, the sampling sites are not comparable since both previous studies collected samples at rooftops, which could explain the absence of tyre wear polymers in the presented results. A summary of previous studies on airborne MNP utilizing active sampling and Py-GC–MS for quantification of plastic and rubber polymers is presented in Table S8.

The results of the present study emphasize the importance of quality assurance protocols in environmental microplastic studies. Based on the laboratory blanks, the selected sample pretreatment protocols resulted in a contamination-free environment. While one of the field blanks showed signals for PE and PP, their proportions of the sample concentrations were minor, on average 17% and 15% for PE and PP, respectively. The instrument was calibrated using pristine polymers mixed with SiO_2_. Environmental MNP are hardly pristine in composition or homogeneous in size, as polymeric particles are exposed to various chemical and physical stressors in the environment, which may modify the polymeric backbone. Toapanta et al. [35] have recently presented that the surface oxidation of PP particles may lead to underestimation of environmental MNP due to the altered composition of generated pyrolysates. In Py-GC–MS analysis, the presence of petroleum products such as automotive exhaust [36], oils and lubricants [37], and bitumen [38, 39] may interfere with the quantitation of PE and PP. Asphalt consists of approximately 95% mineral aggregates, while the remaining 5% includes bitumen and fillers such as thermoplastics and recycled tyres [40, 41]. Additionally, naturally occurring compounds in organic aerosols, i.e. isoprene [42] or natural lipids [43], may cause interferences with NR and PE analysis, respectively. In the present study, evaporation of potentially interfering volatile and semivolatile compounds (e.g. isoprene) was accomplished by heating the sample in a chamber furnace. While the sample pretreatment and PE quantification by 1-eicosene (C20) reduce the interferences from gasoline and diesel, airborne petroleum products with a high boiling point may lead to overestimation of PE. Additionally, pollen has been reported to contain 1–20% of lipids [44], which could lead to overestimation of PE. However, as airborne pollen is generally larger than 10 µm in diameter [45], the effect of pollen on PE quantification in the present study can be considered negligible. These interferences must be considered when analysing total suspended particulate matter or wet and dry deposition. To increase the accuracy of the quantification, sample pretreatment should be as straightforward as possible, and calibration standards should be pretreated as samples to compensate for possible analyte losses during the sample pretreatment.

Preparation of calibration standards in SiO_2_ powder mimicking the sample matrix improves the precision of the quantification of MNP in quartz fibre filter collected samples. Although deuterated polymers have been used as internal standards [46, 47], the organic and inorganic sample matrix may promote deuterium-hydrogen exchange [47], which would result in the overestimation of the corresponding polymers. With the present method, the internal standard recoveries in the PM_10_ samples were 41–166%, 53–153%, and 95–201% of the field blank average for SBR-d14, PI-d7, and PS-d8, respectively (Table S9). When the samples are known to contain a high amount of organic or inorganic components, deuterium-hydrogen exchange must be acknowledged. In such cases, alternative internal standards, such as carbon-13 labelled polymers or polyfluorostyrenes [47], are recommended.

## Conclusions

MNP have been a poorly characterized particle fraction in the atmosphere due to the lack of suitable analysis methods. This study presents a straightforward analytical method suitable for monitoring plastic and rubber polymers in airborne PM samples. Although the presented method was developed and applied for PM_10_ samples, it is applicable to different PM size fractions such as PM_2.5_ or PM_1_. The validation study indicates that airborne MNP mass concentrations can be determined with reasonable measurement uncertainties by Py-GC–MS. In kerbside samples, MNP contributed to 1–3% of PM_10_. These results indicate that MNP mass concentrations should be monitored in health-relevant PM_10_ and PM_2.5_ size fractions at areas with high traffic and population density.

While Py-GC–MS is an advantageous technique for the determination of environmental MNP, quality assurance in the studies must not be neglected. Adequate method validation and quality assurance measures are crucial when interpreting the presented results. Basic research on the thermoanalytical methodology is still necessary to discover polymer-specific pyrolysates and feasible sample pretreatment methods for the variety of plastic and rubber polymers present in the environment. From an analytical point of view, there is an urgent need for certified reference materials with environmentally relevant polymers to enable method validation and intercomparison studies. When publishing quantitative results of environmental MNP, the applied quality assurance protocols and method performance characteristics should be presented to ensure transparency, reproducibility, and comparability of the published results. Accurate quantification of plastic and rubber polymers is a crucial foundation for the studies on sources, transport, distribution, environmental fate, and exposure to micro- and nanoplastic pollution.

## Supplementary Information

Below is the link to the electronic supplementary material.

Supplementary file1 (PDF 1050 KB)

## Data Availability

The authors declare that the data supporting the findings of this study are available within the paper and its Supplementary Information files.

## References

[1] Geyer R, Jambeck JR, Law KL. Production, use, and fate of all plastics ever made. Sci Adv. 2017;3(7): e1700782. 28776036 10.1126/sciadv.1700782 PMC5517107

[2] Plastics Europe - the fast Facts 2024; 2025. Available from: https://plasticseurope.org/knowledge-hub/plastics-the-fast-facts-2024/.

[3] MacLeod M, Arp HPH, Tekman MB, Jahnke A. The global threat from plastic pollution. Science. 2021;373(6550):61–5. 34210878 10.1126/science.abg5433

[4] International Organization for Standardization. ISO 24187:2023 Principles for the analysis of microplastics present in the environment. 2023.

[5] Sommer F, Dietze V, Baum A, Sauer J, Gilge S, Maschowski C, et al. Tire abrasion as a major source of microplastics in the environment. Aerosol Air Qual Res. 2018;18(8):2014–28.

[6] Rafa N, Ahmed B, Zohora F, Bakya J, Ahmed S, Ahmed SF, et al. Microplastics as carriers of toxic pollutants: source, transport, and toxicological effects. Environ Pollut. 2024;343: 123190. 38142809 10.1016/j.envpol.2023.123190

[7] De-la-Torre GE. Microplastics: an emerging threat to food security and human health. J Food Sci Technol. 2020;57(5):1601–8. 32327770 10.1007/s13197-019-04138-1 PMC7171031

[8] Andrady AL. Microplastics in the marine environment. Mar Pollut Bull. 2011;62(8):1596–605. 21742351 10.1016/j.marpolbul.2011.05.030

[9] Li J, Liu H, Paul CJ. Microplastics in freshwater systems: a review on occurrence, environmental effects, and methods for microplastics detection. Water Res. 2018;137:362–74. 29580559 10.1016/j.watres.2017.12.056

[10] Chia RW, Lee J-Y, Kim H, Jang J. Microplastic pollution in soil and groundwater: a review. Environ Chem Lett. 2021;19(6):4211–24.

[11] He D, Luo Y, Lu S, Liu M, Song Y, Lei L. Microplastics in soils: analytical methods, pollution characteristics and ecological risks. TrAC Trends Anal Chem. 2018;109:163–72.

[12] Rillig MC. Microplastic in terrestrial ecosystems and the soil? Environ Sci Technol. 2012;46(12):6453–4. 22676039 10.1021/es302011r

[13] Zarfl C. Promising techniques and open challenges for microplastic identification and quantification in environmental matrices. Anal Bioanal Chem. 2019;411(17):3743–56. 30919016 10.1007/s00216-019-01763-9

[14] Rødland ES, Gustafsson M, Jaramillo-Vogel D, Järlskog I, Müller K, Rauert C, et al. Analytical challenges and possibilities for the quantification of tire-road wear particles. TrAC Trends Anal Chem. 2023;165: 117121.

[15] Luo P, Bai M, He Q, Peng Z, Wang L, Dong C, et al. A novel strategy to directly quantify polyethylene microplastics in PM2.5 based on pyrolysis-gas chromatography–tandem mass spectrometry. Anal Chem. 2023;95(7):3556–62. 10.1021/acs.analchem.2c05477 36757384

[16] Mizuguchi H, Takeda H, Kinoshita K, Takeuchi M, Takayanagi T, Teramae N, et al. Direct analysis of airborne microplastics collected on quartz filters by pyrolysis-gas chromatography/mass spectrometry. J Anal Appl Pyrolysis. 2023;171: 105946.

[17] Morioka T, Tanaka S, Kohama-Inoue A, Watanabe A. The quantification of the airborne plastic particles of 0.43–11 μm: procedure development and application to atmospheric environment. Chemosphere. 2024;351:141131. 10.1016/j.chemosphere.2024.141131 38190942

[18] Chen Y, Jing S, Wang Y, Song Z, Xie L, Shang X, et al. Quantification and characterization of fine plastic particles as considerable components in atmospheric fine particles. Environ Sci Technol. 2024;58(10):4691–703. 38323401 10.1021/acs.est.3c06832

[19] Goßmann I, Herzke D, Held A, Schulz J, Nikiforov V, Georgi C, et al. Occurrence and backtracking of microplastic mass loads including tire wear particles in northern Atlantic air. Nat Commun. 2023;14(1):3707. 37349297 10.1038/s41467-023-39340-5 PMC10287736

[20] International Organization for Standardization. ISO 7708:1995(E) Air quality - particle size fraction definitions for health-related sampling. 1995.

[21] International Organization for Standardization. ISO 23210:2009(en) Stationary source emissions — determination of PM10/PM2,5 mass concentration in flue gas — measurement at low concentrations by use of impactors.

[22] Halonen JI, Lanki T, Yli-Tuomi T, Tiittanen P, Kulmala M, Pekkanen J. Particulate air pollution and acute cardiorespiratory hospital admissions and mortality among the elderly. Epidemiology. 2009;20(1):143. 19234403 10.1097/EDE.0b013e31818c7237

[23] Guaita R, Pichiule M, Maté T, Linares C, Díaz J. Short-term impact of particulate matter (PM2.5) on respiratory mortality in Madrid. Int J Environ Health Res. 2011;21(4):260–74. 21644129 10.1080/09603123.2010.544033

[24] Panko JM, Hitchcock KM, Fuller GW, Green D. Evaluation of tire wear contribution to PM2.5 in urban environments. Atmosphere. 2019;10(2):99.

[25] Giechaskiel B, Grigoratos T, Mathissen M, Quik J, Tromp P, Gustafsson M, et al. Contribution of road vehicle tyre wear to microplastics and ambient air pollution. Sustainability. 2024; 16(2).

[26] Cowger W, Booth AM, Hamilton BM, Thaysen C, Primpke S, Munno K, et al. Reporting guidelines to increase the reproducibility and comparability of research on microplastics. Appl Spectrosc. 2020;74(9):1066–77. 32394727 10.1177/0003702820930292 PMC8216484

[27] European Parliament. Regulation (EU) 2024/1257 of the European Parliament and of the Council of 24 April 2024 on type-approval of motor vehicles and engines and of systems, components and separate technical units intended for such vehicles, with respect to their emissions and battery durability (Euro 7). 2024.

[28] Saarikoski S, Hellén H, Praplan AP, Schallhart S, Clusius P, Niemi JV, et al. Characterization of volatile organic compounds and submicron organic aerosol in a traffic environment. Atmos Chem Phys. 2023;23(5):2963–82.

[29] Dimitrov N, KratofilKrehula L, PtičekSiročić A, Hrnjak-Murgić Z. Analysis of recycled PET bottles products by pyrolysis-gas chromatography. Polym Degrad Stab. 2013;98(5):972–9.

[30] International Organization for Standardization. ISO/TS 20593:2017. Ambient air — determination of the mass concentration of tire and road wear particles (TRWP) — Pyrolysis-GC-MS method. 2017.

[31] Torres-Agullo A, Zuri G, Lacorte S. Pyr-GC-Orbitrap-MS method for the target/untargeted analysis of microplastics in air. J Hazard Mater. 2024;469: 133981. 38461666 10.1016/j.jhazmat.2024.133981

[32] Lauschke T, Dierkes G, Ternes TA. Challenges in the quantification of poly(ethylene terephthalate) microplastics via thermoanalytical methods posed by inorganic matrix components. J Anal Appl Pyrolysis. 2023;174: 106108.

[33] Goßmann I, Süßmuth R, Scholz-Böttcher BM. Plastic in the air?! - Spider webs as spatial and temporal mirror for microplastics including tire wear particles in urban air. Sci Total Environ. 2022;832: 155008. 35381237 10.1016/j.scitotenv.2022.155008

[34] Panko JM, Chu J, Kreider ML, Unice KM. Measurement of airborne concentrations of tire and road wear particles in urban and rural areas of France, Japan, and the United States. Atmos Environ. 2013;72:192–9.

[35] Toapanta T, Okoffo ED, Ede S, O’Brien S, Burrows SD, Ribeiro F, et al. Influence of surface oxidation on the quantification of polypropylene microplastics by pyrolysis gas chromatography mass spectrometry. Sci Total Environ. 2021;796: 148835. 34280630 10.1016/j.scitotenv.2021.148835

[36] Perrone MG, Carbone C, Faedo D, Ferrero L, Maggioni A, Sangiorgi G, et al. Exhaust emissions of polycyclic aromatic hydrocarbons, n-alkanes and phenols from vehicles coming within different European classes. Atmos Environ. 2014;82:391–400.

[37] Kusch P, Obst V, Schroeder-Obst D, Fink W, Knupp G, Steinhaus J. Application of pyrolysis–gas chromatography/mass spectrometry for the identification of polymeric materials in failure analysis in the automotive industry. Eng Fail Anal. 2013;35:114–24.

[38] Nardella F, Duce C, Ribechini E. Analytical pyrolysis and thermal analysis to chemically characterise bitumen from Italian geological deposits and Neolithic stone tools. J Anal Appl Pyrolysis. 2021;158: 105262.

[39] Rødland ES, Samanipour S, Rauert C, Okoffo ED, Reid MJ, Heier LS, et al. A novel method for the quantification of tire and polymer-modified bitumen particles in environmental samples by pyrolysis gas chromatography mass spectroscopy. J Hazard Mater. 2022;423: 127092. 34488093 10.1016/j.jhazmat.2021.127092

[40] Rahman MT, Mohajerani A, Giustozzi F. Recycling of waste materials for asphalt concrete and bitumen: a review. Materials. 2020;13(7):1495. 32218261 10.3390/ma13071495 PMC7177983

[41] Zakerzadeh M, Shahbodagh B, Ng J, Khalili N. The use of waste tyre rubber in Stone Mastic Asphalt mixtures: a critical review. Constr Build Mater. 2024;418: 135420.

[42] Claeys M, Maenhaut W. Secondary organic aerosol formation from isoprene: selected research, historic account and state of the art. Atmosphere. 2021;12(6):728.

[43] Rauert C, Pan Y, Okoffo ED, O’Brien JW, Thomas KV. Extraction and Pyrolysis-GC-MS analysis of polyethylene in samples with medium to high lipid content. J Environ Exp Assess. 2022;1(2):13.

[44] Dahl Å. Pollen lipids can play a role in allergic airway inflammation. Front Immunol. 2018;9. 10.3389/fimmu.2018.02816. 10.3389/fimmu.2018.02816 PMC6297749 30619246

[45] Cuevas-González D, Delgado-Torres JC, Reyna MA, Altamira-Colado E, García-Vázquez JP, Sánchez-Barajas MA, et al. Monitoring of airborne pollen: a patent review. Atmosphere. 2024;15(10):1217.

[46] Unice KM, Kreider ML, Panko JM. Use of a deuterated internal standard with pyrolysis-GC/MS dimeric marker analysis to quantify tire tread particles in the environment. Int. J Enviro. Res Public Health. 2012; 9(11):[4033–55 pp.]. 10.3390/ijerph9114033 PMC3524611 23202830

[47] Lauschke T, Dierkes G, Schweyen P, Ternes TA. Evaluation of poly(styrene-d5) and poly(4-fluorostyrene) as internal standards for microplastics quantification by thermoanalytical methods. J Anal Appl Pyrolysis. 2021;159: 105310.

