# Supplementary material for: Plastic and rubber polymers in urban PM10 by pyrolysis–gas chromatography–mass spectrometry
Source: Anal Bioanal Chem. 2025 May 13;417(17):3835–44. doi: 10.1007/s00216-025-05906-z (PMC12227455; doi:10.1007/s00216-025-05906-z)
Supplement: Supplementary file 1 — Supplementary file1 (PDF 1050 KB) [file 216_2025_5906_MOESM1_ESM.pdf]

Supporting Information for Publication:

Plastic and Rubber Polymers in Urban PM_{10} by Pyrolysis–Gas Chromatography–Mass Spectrometry

Tatu Martinmäki[*1,2], Sanna Saarikoski[3], Hilkka Timonen[3], Jarkko V. Niemi[4], Markus Sillanpää[1]

[1]Finnish Environment Institute, Research Infrastructure, Mustialankatu 3, FI-00790 Helsinki, Finland
[2]University of Helsinki, Department of Chemistry, P.O. BOX 55, FI-00014 Helsinki, Finland
[3]Finnish Meteorological Institute, FI-00560 Helsinki, Finland
[4]Helsinki Region Environmental Services Authority, FI-00520 Helsinki, Finland
[*]Corresponding author

This Supporting Information for Publication contains 11 pages with 1 section, 2 figures and 6 tables.

Table S1: Details of standard and control dilution and cis-polyisoprene calibration and control solutions
Table S2: Blank, standard and aerosol sample data used for quantification of MNPs in kerbside PM_{10} samples
Table S3: Recovery study calibration parameters, results with the corresponding ANOVA tables, and calculated performance characteristics of recovery study
S4: Details of the instrument calibration and blanks
Table S5: Summary of calibration parameters used for quantification of kerbside PM_{10} samples
Figure S6: Calibration curves used for quantification of kerbside PM_{10} samples. Linear calibration curves are presented in black; confidence and prediction intervals are presented in blue and red, respectively.
Figure S7: Example chromatograms for pyrolysates used for quantification or detection (PET) of polymers in kerbside PM_{10} samples
Table S8: Internal standard recoveries of kerbside PM_{10} samples

Table S1: Details of standard and control dilution and cis-polyisoprene calibration and control solutions

Standard dilution		
	Polymer	µg mg^{-1}
	PE	0,8867
	PP	0,2287
	PS	0,0515
	SBR	0,0920
Control dilution		
	Polymer	µg mg^{-1}
	PE	0,8335
	PP	0,2150
	PS	0,0485
	SBR	0,0865
cis-polyisoprene in trichloromethane		
Level	µg/ml	µg
1	2,50	0,0250
2	5,00	0,0500
3	10,0	0,1000
4	20,0	0,2000
5	50,0	0,5000
Control	10,00	0,1000

Table S2: Blank, standard and aerosol sample data used for quantification of MNPs in PM_{10} samples

SAMPLING PERIOD	sample name	filter mass (mg)	subsample mass (mg)	Collection volume (m3)	Polymer mass from py-GC-MS SBR (µg)	PI (µg)	PP (µg)	PET (µg)	PE (µg)	PS (µg)	Results per mg SBR (µg/mg)	PI (µg/mg)	PP (µg/mg)	PET (µg/mg)	PE (µg/mg)	PS (µg/mg)	Results per m3 SBR (µg/m3)	PI (µg/m3)	PP (µg/m3)	PET (µg/m3)	PE (µg/m3)	PS (µg/m3)
	LABORATORY BLANK			0	0,000	0,000	0,000	0,000	0,000	0,000												
	FIELD BLANK 1		8,143	0,11	0,000	0,000	0,000	0,000	0,000	0,000	0,000	0,000	0,000	0,000	0,000	0,000	0,000	0,000	0,000	0,000	0,000	0,000
	FIELD BLANK 2		8,312	0,11	0,000	0,000	0,264	0,000	0,845	0,000	0,000	0,000	0,032	0,000	0,102	0,000	0,000	0,000	0,000	0,000	0,000	0,000
	QFF		10,227	0	0,000	0,000	0,000	0,000	0,000	0,000	0,000	0,000	0,000	0,000	0,000	0,000	0,000	0,000	0,000	0,000	0,000	0,000
	Standard level 1	-	-	-	0,073	0,025	0,180	0,065	0,700	0,041	-	-	-	-	-	-	-	-	-	-	-	-
	Standard level 2	-	-	-	0,149	0,050	0,371	0,134	1,439	0,084	-	-	-	-	-	-	-	-	-	-	-	-
	Standard level 3	-	-	-	0,209	0,100	0,520	0,187	2,016	0,117	-	-	-	-	-	-	-	-	-	-	-	-
	Standard level 4	-	-	-	0,303	0,200	0,752	0,271	2,915	0,170	-	-	-	-	-	-	-	-	-	-	-	-
	Standard level 5	-	-	-	0,477	0,500	1,185	0,427	4,592	0,267	-	-	-	-	-	-	-	-	-	-	-	-
	Standard level 6	-	-	-	0,803	-	1,996	0,720	7,739	-	-	-	-	-	-	-	-	-	-	-	-	-
2.4.2024-9.4.2024	SS_PM10_2 1	102,48	10,445	395,41	0,581	0,224	1,063	2,319	2,091	0,000	0,056	0,021	0,102	0,222	0,200	0,000	0,014	0,006	0,026	0,058	0,052	0,000
	SS_PM10_2 2	102,48	10,566	395,41	0,689	0,236	1,047	2,522	2,267	0,000	0,065	0,022	0,099	0,239	0,215	0,000	0,017	0,006	0,026	0,062	0,056	0,000
	SS_PM10_2 3	102,48	11,709	395,41	0,428	0,270	0,974	8,899	2,017	0,000	0,037	0,023	0,083	0,760	0,172	0,000	0,009	0,006	0,022	0,197	0,045	0,000
9.4.2024-15.4.2024	SS_PM10_4 1	117,45	13,304	322,6	0,999	0,626	1,374	4,248	2,825	0,052	0,075	0,047	0,103	0,319	0,212	0,004	0,027	0,017	0,038	0,116	0,077	0,001
	SS_PM10_4 2	117,45	13,732	322,6	1,059	0,748	1,648	2,814	3,515	0,067	0,077	0,054	0,120	0,205	0,256	0,005	0,028	0,020	0,044	0,075	0,093	0,002
	SS_PM10_4 3	117,45	12,476	322,6	0,990	0,688	2,089	5,943	2,256	0,047	0,079	0,055	0,167	0,476	0,181	0,004	0,029	0,020	0,061	0,173	0,066	0,001
15.4.2024-18.4.2024	SS_PM10_5 1	114,17	11,186	341,98	1,004	0,739	2,478	1,947	5,153	0,042	0,090	0,066	0,222	0,174	0,461	0,004	0,030	0,022	0,074	0,058	0,154	0,001
	SS_PM10_5 2	114,17	13,769	341,98	1,101	0,977	1,748	2,301	3,949	0,025	0,080	0,071	0,127	0,167	0,287	0,002	0,027	0,024	0,042	0,056	0,096	0,001
	SS_PM10_5 3	114,17	14,046	341,98	2,001	1,052	1,435	0,864	3,773	0,014	0,142	0,075	0,102	0,061	0,269	0,001	0,048	0,025	0,034	0,021	0,090	0,000
22.4.2024-2.5.2024	SS_PM10_7 1	121,12	11,585	546,46	5,905	2,320	3,660	7,758	6,999	0,252	0,510	0,200	0,316	0,670	0,604	0,022	0,113	0,044	0,070	0,148	0,134	0,005
	SS_PM10_7 2	121,12	10,844	546,46	4,971	1,898	2,746	13,629	6,796	0,197	0,458	0,175	0,253	1,257	0,627	0,018	0,102	0,039	0,056	0,279	0,139	0,004
	SS_PM10_7 3	121,12	12,48	546,46	5,787	2,433	4,682	1,260	10,703	0,245	0,464	0,195	0,375	0,101	0,858	0,020	0,103	0,043	0,083	0,022	0,190	0,004
6.5.2024-20.5.2024	SS_PM10_8 1	124,63	10,436	770,48	5,789	2,231	2,158	11,218	4,961	0,366	0,555	0,214	0,207	1,075	0,475	0,035	0,090	0,035	0,033	0,174	0,077	0,006
	SS_PM10_8 2	124,63	10,334	770,48	5,969	2,666	1,502	7,095	3,736	0,259	0,578	0,258	0,145	0,687	0,362	0,025	0,093	0,042	0,024	0,111	0,058	0,004
	SS_PM10_8 3	124,63	9,889	770,48	4,552	2,309	1,328	5,950	3,440	0,292	0,460	0,234	0,134	0,602	0,348	0,030	0,074	0,038	0,022	0,097	0,056	0,005
20.5.2024-3.6.2024	SS_PM10_9 1	123,59	11,822	763,63	6,337	2,239	2,873	35,988	11,479	0,241	0,536	0,189	0,243	3,044	0,971	0,020	0,087	0,031	0,039	0,493	0,157	0,003
	SS_PM10_9 2	123,59	12,373	763,63	6,143	2,186	3,102	58,853	13,047	0,274	0,497	0,177	0,251	4,757	1,054	0,022	0,080	0,029	0,041	0,770	0,171	0,004
	SS_PM10_9 3	123,59	13,134	763,63	6,828	2,329	3,123	45,389	15,411	0,253	0,520	0,177	0,238	3,456	1,173	0,019	0,084	0,029	0,038	0,559	0,190	0,003

Table S3: Recovery study calibration parameters, results with the corresponding ANOVA tables, and calculated performance characteristics of recovery study

	**Calibration parameters**					
**Polymer**	**R^2 day 1**	**R^2 day 2**	**R^2 day 3**	**f(x) day 1**	**f(x) day 2**	**f(x) day 3**
PE	0.939	0.967	0.915	0.024946*x-0.004539	0.025987*x-0.000957	0.021720*x-0.010673
PP	0.910	0.978	0.956	f(x)=0.175318*x-0.005111	0.188432*x+0.002273	0.179184*x-0.024248
PS	0.990	0.907	0.950	f(x)=1.803060*x+0.014920	1.913252*x+0.043866	1.857885*x-0.003315
SBR	0.947	0.943	0.986	f(x)=1.423312*x+0.086282	1.262082*x+0.103407	1.601073*x+0.029164
NR	0.939	0.984	0.971	f(x)=0.264443*x-0.000557	1.262082*x+0.103407	0.305995*x+0.001809
**Recovery study results and corresponding Anova tables**						
**PE**						
replicate	day 1 (µg/mg)	day 2 (µg/mg)	day 3 (µg/mg)			
1	0,777	0,863	0,913			
2	0,809	1,071	1,046			
3	0,766	1,342	0,836			
4	0,915	0,858	0,614			
5	0,665		0,618			
6			1,024			
Anova: Single Factor						
SUMMARY						
Groups	Count	Sum	Average	Variance		
series 1	5	3,9323	0,7865	0,0080		
series 2	4	4,1337	1,0334	0,0521		
series 3	6	5,0503	0,8417	0,0364		
ANOVA						
Source of Variation	SS	df	MS	F	P-value	F crit
Between Groups	0,146241	2	0,07312	2,368822	0,135807	3,885294
Within Groups	0,370413	12	0,030868			
Total	0,516654	14				
**PP**						
replicate	day 1 (µg/mg)	day 2 (µg/mg)	day 3 (µg/mg)			
1	0,230	0,219	0,187			
2	0,211	0,265	0,202			
3	0,202	0,408	0,185			
4	0,227	0,225	0,156			
5	0,156		0,170			

6			0,233			
Anova: Single Factor						
SUMMARY						
Groups	Count	Sum	Average	Variance		
series 1	5	1,0253	0,2051	0,0009		
series 2	4	1,1174	0,2793	0,0077		
series 3	6	1,1343	0,1891	0,0007		
ANOVA						
Source of Variation	SS	df	MS	F	P-value	F crit
Between Groups	0,020913	2	0,010457	4,126046	0,043278	3,885294
Within Groups	0,030412	12	0,002534			
Total	0,051325	14				
**PS**						
replicate	day 1 (µg/mg)	day 2 (µg/mg)	day 3 (µg/mg)			
1	0,037	0,035	0,038			
2	0,043	0,042	0,053			
3	0,056	0,063	0,050			
4	0,062	0,064	0,054			
5	0,055		0,054			
6			0,050			
Anova: Single Factor						
SUMMARY						
Groups	Count	Sum	Average	Variance		
series 1	5	0,2537	0,0507	0,0001		
series 2	4	0,2037	0,0509	0,0002		
series 3	6	0,2981	0,0497	0,0000		
ANOVA						
Source of Variation	SS	df	MS	F	P-value	F crit
Between Groups	4,67E-06	2	2,33E-06	0,022978	0,977327	3,885294
Within Groups	0,001219	12	0,000102			
Total	0,001224	14				
**SBR**						
replicate	day 1 (µg/mg)	day 2 (µg/mg)	day 3 (µg/mg)			

1	0,081	0,100	0,081			
2	0,097	0,086	0,097			
3	0,104	0,083	0,104			
4	0,093	0,087	0,093			
5	0,088		0,088			
6			0,081			
Anova: Single Factor						
SUMMARY						
Groups	Count	Sum	Average	Variance		
series 1	5	0,4625	0,0925	0,0001		
series 2	4	0,3568	0,0892	0,0001		
series 3	6	0,5434	0,0906	0,0001		
ANOVA						
Source of Variation	SS	df	MS	F	P-value	F crit
Between Groups	2,50E-05	2	1,25E-05	0,166243	0,848758	3,885294
Within Groups	0,000904	12	7,53E-05			
Total	0,000929	14				
**NR**						
replicate	day 1 (µg/mg)	day 2 (µg/mg)	day 3 (µg/mg)			
1	0,086	0,086	0,107			
2	0,086	0,097	0,110			
3	0,095	0,121	0,095			
4	0,110	0,099	0,086			
5	0,107		0,086			
6			0,099			
Anova: Single Factor						
SUMMARY						
Groups	Count	Sum	Average	Variance		
series 1	5	0,4839	0,0968	0,0001		
series 2	4	0,4029	0,1007	0,0002		
series 3	6	0,5832	0,0972	0,0001		
ANOVA						
Source of Variation	SS	df	MS	F	P-value	F crit
Between Groups	4,06E-05	2	2,03E-05	0,143223	0,86802	3,885294
Within Groups	0,001701	12	0,000142			

Total	0,001742	14				
**Method performance characteristics**						
	**Bias-%**	**s_I-%**	**u_c (%)**	**U (%)**		
PE	4,91	24,24	24,73	50		
PP	1,61	32,92	32,96	70		
PS	3,85	23,03	23,35	50		
SBR	5,02	10,79	11,91	25		
NR	-2	13,77	13,91	30		

S4: Details of the instrument calibration and blanks

Calibration of the instrument prior to the analysis of the samples showed linearity with coefficients of determination R^2 > 0.9 for each of the pyrolysates, Details of the instrument calibration parameters are presented in Table S5. Calibration curves with confidence and prediction factors are presented in Figure S6. Quantification of the polymers in kerbside aerosol samples showed good reproducibility, which indicates successful homogenization of the sample (RSD-% 3–70%; n=3). Out of the two field blanks analysed, Field blank 1 (collected before April 2nd) was negative for each of the studied polymers, while Field blank 2 (collected before April 22nd) showed a signal above the LLOQ for PE and PP. The calculated signal accounted for an average of 17% and 15% of the PE and PP mass, respectively, in the samples collected after April 22nd. Field blank 2 concentrations have been subtracted from aerosol samples collected after April 22nd. No signals were detected in the laboratory and instrument blanks for the studied polymers, indicating no interfering contamination from the sample pretreatment or the instrument, respectively. Example chromatograms of the studied pyrolysates in aerosol samples are presented in Figure S7.

Table S5: Summary of calibration parameters used for quantification of kerbside PM_{10} samples

Pyrolysate (plastic/rubber)	LLOQ (µg)	HLOQ (µg)	fit	points	R^2	f(x)=	weight
4-vinylcyclohexene (SBR/BR)	0.073	0.803	linear	6	0.983	1.178861*x+0.109496	1/x
Dipentene (NR)	0.025	0.500	linear	5	0.996	0.348582*x+0.003847	none
2,4,6-trimethyl-1-nonene (PP)	0.180	1.996	linear	6	0.976	0.194842*x-0.022478	1/x
1-eicosene (PE)	0.700	7.739	linear	6	0.966	0.031047*x-0.011290	$1/x^2$
2,4,6-triphenyl-1-hexene (PS)	0.041	0.267	linear	5	0.902	1.182827*x+0.081098	$1/x^2$

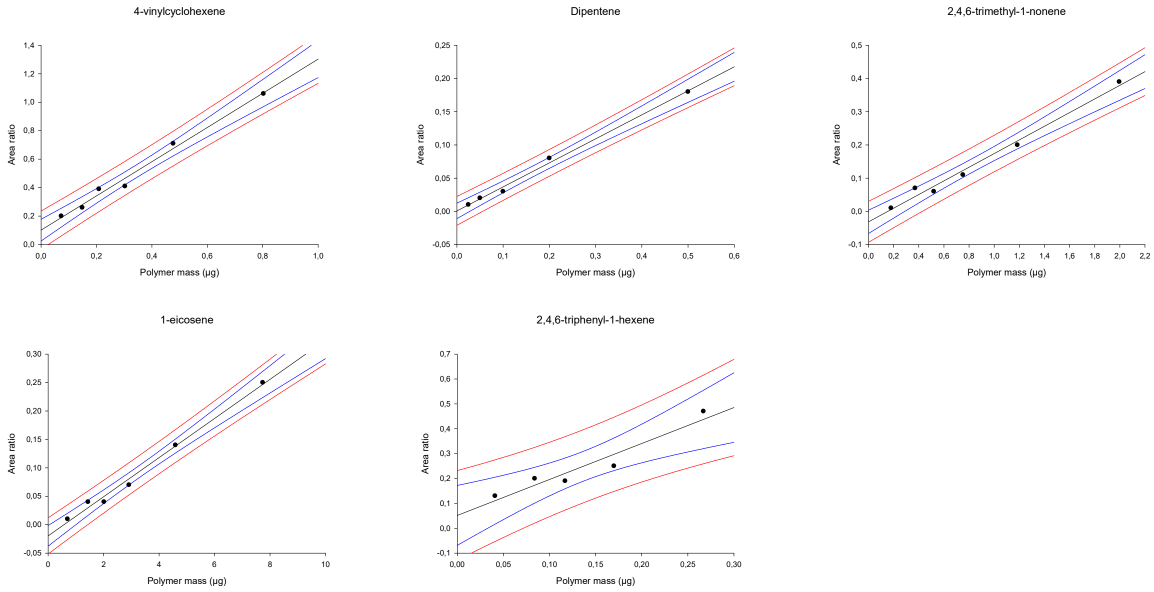

Figure S6: Calibration curves used for quantification of PM_{10} samples. Linear calibration curves are presented in black; confidence and prediction intervals are presented in blue and red, respectively.

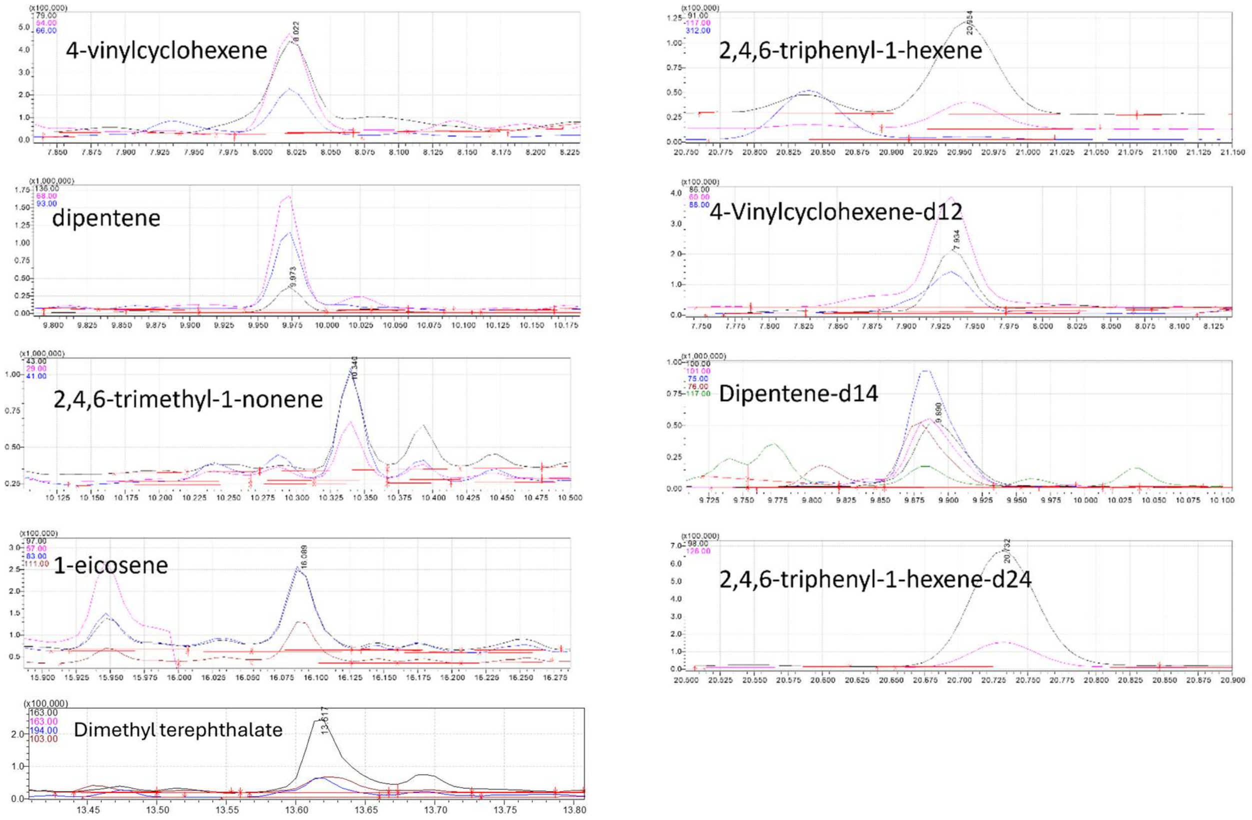

Figure S7: Example chromatograms for pyrolysates used for quantification or detection (PET) of polymers in aerosol samples.

Table S8: Summary of previous studies on airborne MNP utilizing active sampling and Py-GC-MS

**Study**	**Size fraction studied**	**Polymers detected in aerosol samples**	**Internal standard(s)**	**Quality assurance**	**Total polymer mass concentrations in airborne particulate matter**
Chen et al. (2024)	$PM_{2.5}$	PE, PS, PVC	none	Blanks, sample processing on	0-24 µg m^{-3}; average 6 µg m-3

				clean bench. No validation data presented.	
Goßmann et al. (2023)	>10 µm, 5-10 µm	PE, PET, PP, PS, PC, PA, PU, and car and truck tyre treads	deuterated PS	Blanks, sample processing on clean bench. No validation data presented.	0,2-0,5 ng m^{-3} in low volume samples
Luo et al. (2023)	$PM_{2.5}$	PE	Dodecane	Blanks. Recovery study for repeatability of the method, LODs calculated from blank samples.	0.8–13.7 pg m^{-3}
Mizuguchi et al. (2023)	$>PM_{10}$, $PM_{2.5\text{-}10}$, $PM_{2.5}$	PP, PS, SBR	none	none	1.3–1.9 ng m^{-3} $PM_{2.5–10}$
Morioka et al. (2024)	0.43–11 µm	PE, PET, PS, PA66,	none	Blanks, sample processing on clean bench. IDL determined for studied polymers.	980 ng m^{-3} for size fraction < 11 µm
Martinmäki et al (present study, 2025)	PM_{10}	PE, PET, PP, PS, NR, SBR	deuterated PS, deuterated SBR, deuterated NR/cis-polyisoprene)	Blanks, sample processing on clean bench. Linear range, LOQs, intermediate precision and bias, and measurement uncertainties determined in a validation study.	96–350 ng m^{-3}

Table S9: Internal standard recoveries of PM10 samples

4-vinylcyclohexene-d12					
Data Filename	Sample Name	Ret. Time	Area	Height	%-Field blank average
20241806_013.qgd	SS_PM10_3 BL (Field blank 1)	7,931	462626	187,586	
20241806_014.qgd	SS_PM10_6 BL (Field blank 2)	7,927	485924	207,352	
-	Field blank average		474275		100
20241806_015.qgd	QFF	7,927	394037	167,7	83
20241906_004.qgd	SS_PM10_2 1	7,93	352872	140,366	74
20241906_008.qgd	SS_PM10_4 1	7,931	539509	175,32	114
20241906_012.qgd	SS_PM10_2 2	7,934	427309	174,743	90
20241906_013.qgd	SS_PM10_2 3	7,936	604081	175,236	127
20241906_016.qgd	SS_PM10_4 2	7,942	624298	190,06	132
20241906_017.qgd	SS_PM10_4 3	7,946	634724	206,681	134
20242006_005.qgd	SS_PM10_5 1	7,925	787000	270,475	166
20242006_006.qgd	SS_PM10_5 2	7,938	777034	195,085	164
20242006_007.qgd	SS_PM10_5 3	7,933	414631	169,8	87
20242006_011.qgd	SS_PM10_7 1	7,931	393466	202,229	83
20242006_012.qgd	SS_PM10_7 2	7,932	459028	247,916	97
20242006_013a.qgd	SS_PM10_7 3	7,95	270923	113,668	57

20242006_017.qgd	0+is (laboratory blank)	7,932	225523	90,144	48
20242006_018.qgd	SS_PM10_8 1	7,931	228641	115,754	48
20242006_019.qgd	SS_PM10_8 2	7,939	193298	80,117	41
20242006_020.qgd	SS_PM10_8 3	7,957	207148	95,902	44
20242006_024.qgd	SS_PM10_9 1	7,953	250599	96,221	53
20242006_025.qgd	SS_PM10_9 2	7,949	300539	133,792	63
20242006_026.qgd	SS_PM10_9 3	7,949	291058	110,585	61
dipentene-d14					
Data Filename	Sample Name	Ret. Time	Area	Height	%-Field blank average
20241806_013.qgd	SS_PM10_3 BL (Field blank 1)	9,88	158229	575,366	
20241806_014.qgd	SS_PM10_6 BL (Field blank 2)	9,879	180209	651,161	
	Field blank average		169219		100
20241806_015.qgd	QFF	9,879	141268	510,153	83
20241906_004.qgd	SS_PM10_2 1	9,879	130926	479,622	77
20241906_008.qgd	SS_PM10_4 1	9,882	152479	554,554	90
20241906_012.qgd	SS_PM10_2 2	9,885	157968	568,75	93
20241906_013.qgd	SS_PM10_2 3	9,887	164478	604,929	97
20241906_016.qgd	SS_PM10_4 2	9,89	165691	602,865	98
20241906_017.qgd	SS_PM10_4 3	9,89	178201	647,83	105
20242006_005.qgd	SS_PM10_5 1	9,884	177468	634,6	105
20242006_006.qgd	SS_PM10_5 2	9,887	157597	570,284	93
20242006_007.qgd	SS_PM10_5 3	9,889	142266	513,76	84
20242006_011.qgd	SS_PM10_7 1	9,89	197777	699,86	117
20242006_012.qgd	SS_PM10_7 2	9,892	259568	917,495	153
20242006_013a.qgd	SS_PM10_7 3	9,892	102905	376,743	61
20242006_017.qgd	0+is (laboratory blank)	9,885	88848	311,344	53
20242006_018.qgd	SS_PM10_8 1	9,885	145880	517,256	86
20242006_019.qgd	SS_PM10_8 2	9,888	103293	371,834	61
20242006_020.qgd	SS_PM10_8 3	9,895	117930	414,985	70
20242006_024.qgd	SS_PM10_9 1	9,896	138196	487,942	82
20242006_025.qgd	SS_PM10_9 2	9,894	176296	629,793	104
20242006_026.qgd	SS_PM10_9 3	9,897	157470	520,54	93
2,4,6-triphenyl-1-hexene-d24					
Data Filename	Sample Name	Ret. Time	Area	Height	%-Field blank average
20241806_013.qgd	SS_PM10_3 BL (Field blank 1)	20,719	148331	412,911	
20241806_014.qgd	SS_PM10_6 BL (Field blank 2)	20,719	175355	488,626	
	Field blank average		161843		100
20241806_015.qgd	QFF	20,721	152169	418,308	94
20241906_004.qgd	SS_PM10_2 1	20,715	258574	744,529	160
20241906_008.qgd	SS_PM10_4 1	20,721	290888	841,442	180
20241906_012.qgd	SS_PM10_2 2	20,728	265010	768,066	164
20241906_013.qgd	SS_PM10_2 3	20,728	312575	899,936	193

20241906_016.qgd	SS_PM10_4 2	20,732	324563	941,491	201
20241906_017.qgd	SS_PM10_4 3	21,198	262515	760,806	162
20242006_005.qgd	SS_PM10_5 1	20,76	171713	490,194	106
20242006_006.qgd	SS_PM10_5 2	20,765	270141	779,718	167
20242006_007.qgd	SS_PM10_5 3	20,732	252240	721,912	156
20242006_011.qgd	SS_PM10_7 1	20,82	272878	783,565	169
20242006_012.qgd	SS_PM10_7 2	20,738	268956	747,909	166
20242006_013a.qgd	SS_PM10_7 3	20,73	154161	438,044	95
20242006_017.qgd	0+is (laboratory blank)	20,835	253596	740,688	157
20242006_018.qgd	SS_PM10_8 1	20,886	238046	655,783	147
20242006_019.qgd	SS_PM10_8 2	20,816	283932	779,368	175
20242006_020.qgd	SS_PM10_8 3	20,956	307003	831,802	190
20242006_024.qgd	SS_PM10_9 1	20,986	250889	654,289	155
20242006_025.qgd	SS_PM10_9 2	20,928	212609	547,769	131
20242006_026.qgd	SS_PM10_9 3	20,946	237832	611,1	147
